# Tick-Tock: Cancer Cell Division Cycle Clocks Strike Midnight

**DOI:** 10.3390/ijms26136274

**Published:** 2025-06-29

**Authors:** Scott C. Schuyler, Hsin-Yu Chen, Tran Thi Bao Nguyen, Cheng-Ye Weng, Katelyn Huang, Yun-Chen Renee Lin

**Affiliations:** 1 Department of Biomedical Sciences, College of Medicine, Chang Gung University, Kwei-Shan, Taoyuan 333, Taiwan; 2 Department of Otolaryngology—Head and Neck Surgery, Chang Gung Memorial Hospital, Taoyuan 333, Taiwan

**Keywords:** cancer, proteotoxic stress, cell division cycle, heat-shock protein 90 (HSP90), anaphase-promoting complex/cyclosome (APC/C)

## Abstract

Eukaryotic cells double their mass and divide at the same rate, allowing cells to maintain a uniform cell size over many cell divisions. We hypothesize that aneuploid cancer cells are more sensitive to forced overgrowth, more than doubling their mass during a single longer-duration cell division cycle, relative to healthy diploid cells. This hypothesis stems from the observation that cancer cells are under proteotoxic stress, during which heat-shock proteins become rate-limiting and the unfolded-protein response network has a growth-suppressive phenotype. Forced overgrowth will lead to the production of more individual proteins per cell division cycle and increase the duration of time during which any mis-folded or aggregated proteins might disrupt the function of properly folded proteins. To induce these potential forced overgrowth effects, we suggest targeting the cell division cycle regulatory enzyme, the anaphase-promoting complex/cyclosome (APC/C), to suppress—but not inhibit—its activity. We conclude by proposing experiments to test this hypothesis in which an APC/C inhibitor, such as a low level of proTAME, is combined with the clinically approved heat-shock protein 90 (HSP90)-inhibitor pimitespib (TAS-116) or the pre-clinical molecule tanespimycin, which, to the best of our knowledge, are combinations that have not been investigated before.

## 1. Introduction

Aneuploidy is a strong and unique feature of dividing cancer cells [1,2]. There is no healthy dividing human cell population that is aneuploid as part of its normal genetically programmed lifespan. Non-dividing aneuploid cells do exist naturally in the human central nervous systems, for example, and humans also harbor populations of polyploid dividing cells, but there are no known genetically pre-programmed aneuploid dividing cells. Thus, this feature is not only prevalent within cancer cells, but it is also unique to cancer cells as a diseased cellular state. Our goal is to explore what therapeutic opportunity this unique cellular condition of cancer cells might create by focusing on the proteotoxic stress induced by cancer cell aneuploidy and how it might be leveraged indirectly by manipulating the duration of the cell division cycle to force the overgrowth of cancer cells and promote cancer cell death [2,3,4].

## 2. Cancer Cell Proteotoxic Stress and Aneuploidy

**Figure 1:**
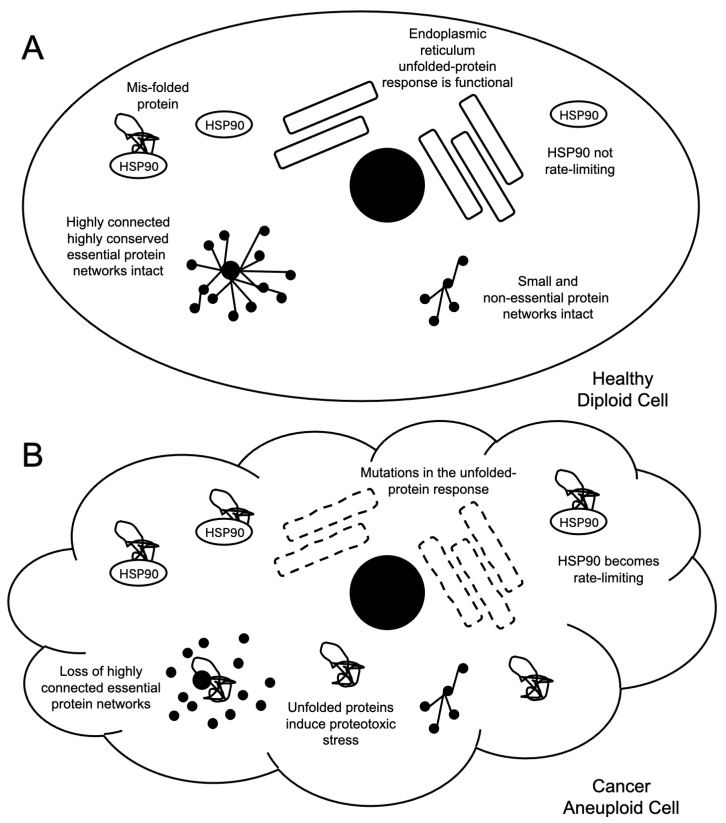
Cancer cell aneuploidy induces proteotoxic stress. (**A**) In healthy cells, HSP90 is not rate-limiting, the unfolded-protein response is functional, and essential networks, denoted by the highly connected black dots, remain intact and functional. (**B**) Cancer cells display proteotoxic stress. Stoichiometric imbalances saturate HSP90 with unfolded proteins. Aneuploidy selects for mutants in the unfolded-protein response system that releases cells from growth suppression. A loss of HSP90 function does not affect all cellular pathways equally; rather, essential, highly conserved, and highly connected proteins in dense protein networks are the most vulnerable to HSP90 depletion.

One major consequence of cells becoming aneuploid is the resultant proteotoxic stress [2,5] (Figure 1). This stress is thought to be derived from three primary sources: First, aneuploidy leads to measured stoichiometric imbalances in the expression levels of the protein components of multi-protein complexes, causing essential protein chaperones, such as heat-shock protein 90 (HSP90), to become limiting for viability in cells [2,5,6]. This limitation likely emerges from a loss of buffering capacity, namely, an ability to tolerate a natural load on the protein-folding machinery in the cell. Such capacity is critical for the normal and routine assembly and maintenance of multiprotein complexes, especially large complexes or proteins that have many different binding partners in the cell, where one established example is the anaphase-promoting complex/cyclosome (APC/C) [7,8]. Buffering capacity is a fundamental cell biological function necessary for cell viability and is a core requisite function to achieve proper cellular proteostasis. Cancer cells are likely to become more dependent on these buffering capacity systems as they develop aneuploidy, which is a weakness that may have the potential to be exploited therapeutically [2,5,6].

Second, aneuploidy in eukaryotic cells leads to mutations in genes encoding unfolded-protein response proteins as they limit the rates of cell division [9]. In the model system budding yeast, when artificial aneuploid cells were engineered, the resultant cells displayed a slow-growth phenotype that could be suppressed by mutations in the unfolded-protein response network. This implies that proper protein folding contributes to the duration of the cell division cycle, where proper protein folding creates a load on the cellular machinery, slowing cell cycle progression. Such a load slowing the rate of proliferation may be selected against during tumorigenesis. However, the loss of the unfolded-protein response pathway, like a decrease or loss of protein folding buffering in general, may create a cancer cell specific vulnerability.

Third, when HSP90 becomes limited in cells, not all cellular networks are disrupted equally, and, in fact, the highly connected and highly conserved cellular processes that are essential for cell viability display a higher vulnerability to this injury [10]. An individual unfolded protein in a cell will randomly encounter other proteins. Such an encounter may lead to a loss of function in the protein that the unfolded protein randomly encounters. However, it appears that not all proteins share an equal sensitivity to the presence of unfolded proteins in the cell [10]. Proteins that tend to be highly conserved and essential for cell viability appear to be more vulnerable as they tend to be highly connected within protein–protein interaction networks [10]. As noted above, the APC/C is one such example [7,8]. As such, not all random encounters with an unfolded protein are equal—and those that occur with the conserved, essential, and highly connected proteins are the ones that will increase the risk of cell death occurring as a result of the random interaction.

Exploiting proteotoxic stress directly, by targeting the mechanisms that help cells respond to and recover from such stress, has long been the target of anti-cancer therapeutics [2,3,11,12]. This has resulted in several anti-cancer successes including Food and Drug Administration (FDA)-approved 26S proteosome inhibitors such as bortezomib, carfilzomib, and ixazomib; the Histone Deacetylase 6 (HDAC6) inhibitor panobinostat; and pimitespib (TAS-116), an HSP90 inhibitor approved for use against stomach cancer in Japan [3]. These successes validate that proteotoxic stress is truly a challenge for cancer cells that can be exploited therapeutically. However, there have been limitations too, such as non-specific cytotoxicity and off-target effects as the mechanisms involved in responding to cancer cell proteotoxicity are part of widely used protein homeostasis pathways [3]. Therefore, it may be useful to explore ways in which this known vulnerability, namely, proteotoxic stress, could be exploited further, perhaps in an indirect manner, to potentiate current chemotherapeutic drugs or pre-clinical anti-cancer small molecules by trying to elevate the effects of aneuploidy-induced proteotoxicity.

## 3. Cell Growth and the Cell Division Cycle as a Therapeutic Target

**Figure 2:**
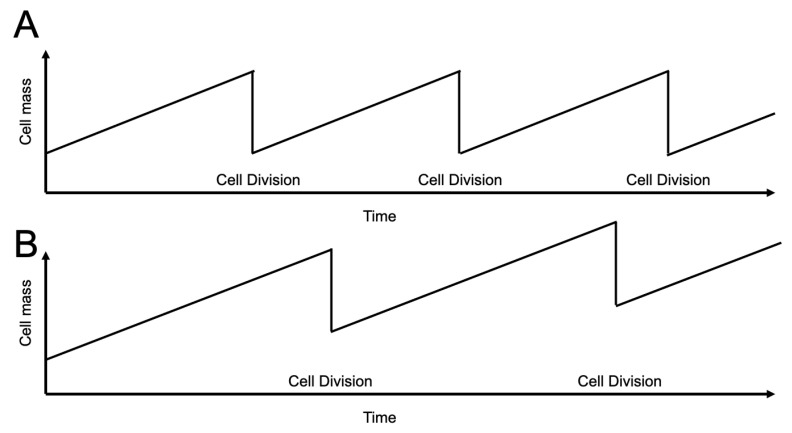
The rate of cell growth and frequency of the cell division cycle are coupled. (**A**) Cells remain roughly the same size throughout many rounds of cell division. (**B**) Our strategy is to break this relationship by manipulating the cell division cycle machinery to force overgrowth during each cell division cycle.

It is well established that the ratio between the amount of protein a eukaryotic cell produces is directly proportional to cell growth and that the rate of cell growth (i.e., total protein production) is coupled in most cells to the frequency of cell division [4]. The amount of protein produced per individual cell division cycle is nearly equal to that at the beginning of the cell division cycle in the early G1-phase so that cells remain roughly the same size throughout subsequent rounds of cell division (Figure 2).

We propose that cancer cells may be more sensitive to wastefully overgrow—i.e., to being forced to overproduce the amount of proteins that they need during one cell division cycle by delaying cell cycle progression (Figure 2). The concept is that during the execution of each longer-duration individual cell division cycle, the cell mass will continue to increase and increase with each subsequent division. The resultant cell divisions will occur less frequently over time, suppressing proliferation, and will also increase the total amount of protein per cell division cycle each cell produces—including, in aneuploid cells, an increase in the number of unfolded proteins per cell—essentially causing the cells to metabolize themselves to death by over producing unnecessary levels of toxic unfolded proteins in the context of being aneuploid during a single longer-duration cell division cycle.

**Figure 3:**
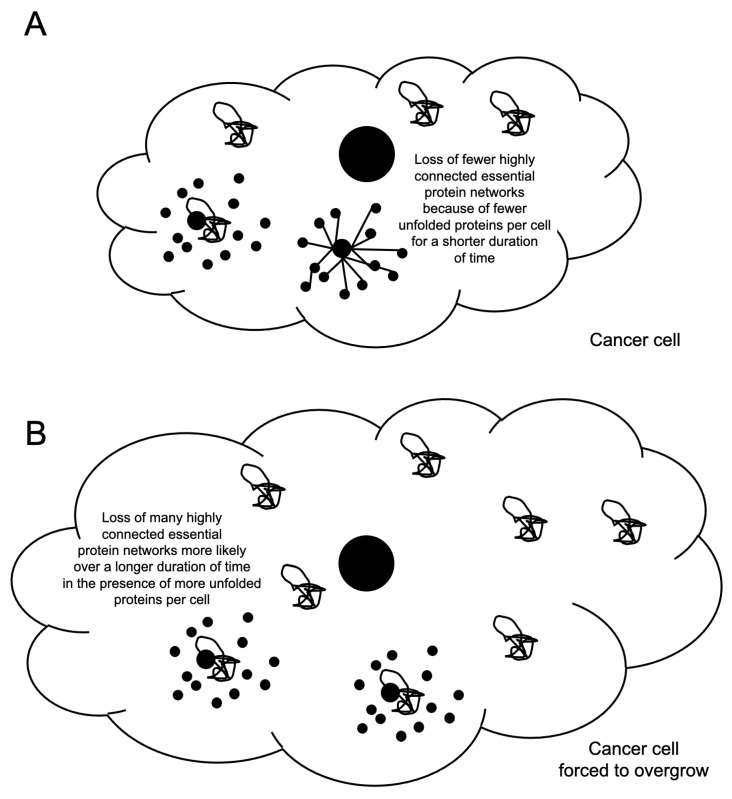
Forced overgrowth, by extending the duration of the cell division cycle, may enhance the effects of proteotoxic stress. (**A**) For comparison, a cancer cell with an unmanipulated cell division cycle may retain functional essential protein networks, as denoted by highly connected black dots, and viability. (**B**) If cancer cells produce more protein per single cell division cycle, this will likely increase the level of unfolded proteins per individual cell. Although the molar concentration of unfolded proteins per volume may not change, the increased number of unfolded proteins will increase the likelihood that essential highly conserved protein networks will become dysfunctional, indicated by a loss of connections between the dots, leading to cell death. Extending the cell division cycle duration will increase the amount of time per individual cell that an unfolded protein can disrupt and damage an essential protein network, leading to cell death.

This manipulation of the cell cycle is, of course, likely to be very stressful for healthy human cells too, but we speculate that due to the prevalence of aneuploidy that only exists in dividing cancer cells, these stresses will be less severe in healthy normal diploid cells and more lethal to aneuploid cancer cells that are under constant proteotoxic stress (Figure 3). Here, with regard to cytotoxicity, we are encouraged by the past positive results with the successful development of G1-cyclin-Cyclin-Dependent Kinase (CDK) inhibitors targeting the CDK4/6 kinases such as palbociclib, ribociclib, and abemaciclib, which have been proposed to suppress cancer cell proliferation by stopping cell cycle progression at the G1-S-phase transition without displaying such severe side effects [13]. These agents are thought to act by prolonging the duration of the G1-phase of the cell cycle and may not actually promote cell death on their own [14]. They are used in combination with other anti-cancer agents in the treatment of a variety of cancers.

We further suspect that forced overgrowth is likely to display an even more severe cytotoxic effect, especially in combination with the stress challenges cancer cells are under when exposed to established chemotherapeutic agents, such as paclitaxel (see below). If we can force aneuploid dividing cells, which are only ever present in humans as cancer cells, to produce more proteins per cell division cycle and/or take longer to traverse a single cell division cycle, this may increase the potential lethality of each individual unfolded protein that they produce. The more proteins produced in a single cell division cycle under proteotoxic stress will increase the probability that a newly mis-folded protein will disrupt and cause dysfunction in an essential protein network [10]. The longer a mis-folded protein has to disrupt protein networks, the more likely it is to disrupt an essential protein network. In combination, the disruption of any essential network in a cell, especially in the context of a cancer cell that has already been exposed to a chemotherapeutic agent, is likely, we argue, to elevate the levels of cancer cell death.

This raises the following question: what is the best cell division cycle component to target in order to force cancer cells to overgrow and potentially enhance the proteotoxic stress they are under? A critical corollary to this question is how to extend the duration of the cell division cycle without blocking cell division cycle progression altogether. A complete block of the cell division cycle would surely be a lethal toxic event to healthy human dividing cells as well. Furthermore, the cell division cycle is a highly robust complex system that is difficult to disrupt as robustness is a feature of a system’s ability to resist any perturbation. Robustness means that it takes a strong action to perturb the system away from its equilibrium, and, even if the system is pushed away from the equilibrium point, by design, it will very quickly re-equilibrate itself and remain functional.

**Figure 4:**
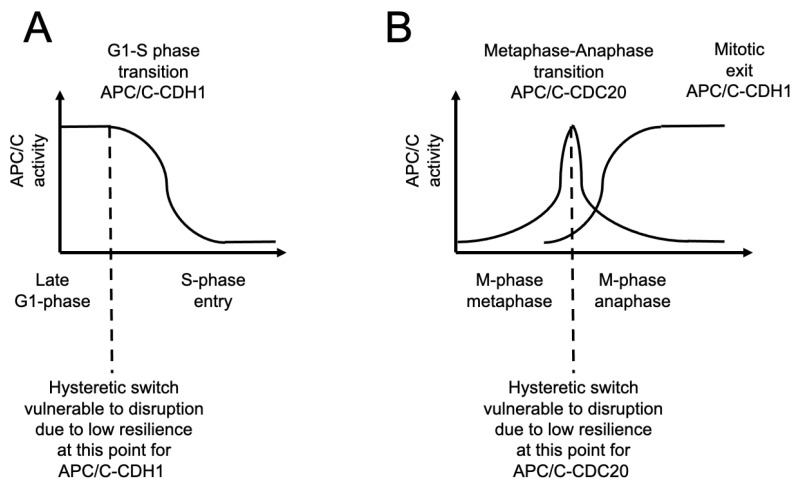
Major cell cycle transitions that occur via hysteresis may be vulnerable points in the cell division cycle. Illustrated here are cell cycle transitions that are catalyzed in part by the master cell cycle regulatory enzyme the anaphase-promoting complex/cyclosome (APC/C) using different co-factors, either CDH1 or CDC20. (**A**) One major cell cycle transition is START, namely, the G1-S-phase transition at which point APC/C-CDH1 activity is required to allow for the initiation of S-phase and DNA replication. (**B**) A second major cell division cycle transition is the metaphase–anaphase transition which requires the activity of APC/C-CDC20 to promote the destruction of securin, an inhibitor of anaphase entry. Once cells enter anaphase, APC/C-CDH1 becomes active and promotes mitotic exit.

Yet, there are vulnerable moments in the cell division cycle system that we may take advantage of, namely, the cell cycle transitions. Two of the most prominent cell cycle transitions are the G1-S-phase transition and the metaphase–anaphase transition in mitosis (M-phase) (Figure 4). Both have been shown to display hysteresis and bistability in an evolutionarily conserved manner [4]. It is known that when a hysteretic switch approaches a transition point in its complex system equilibrium, it displays a phenomenon called “critical slowing-down”, a system state during which the system as a whole becomes very vulnerable to even very minor perturbations and is said to lose “resilience”. A loss of resilience in a robust system specifically means that once the complex system is disrupted in this vulnerable “critical slowing-down” moment, even by a minor disruption, it will take the system a long time to re-equilibrate before it can execute the transition. The system does not break down but rather simply suffers a delay in forward progression as it re-equilibrates. It is this long duration of recovery we seek to take advantage of to force cancer cells to overgrow and kill themselves by producing excess unfolded proteins per each cell division cycle.

## 4. Targeting the APC/C to Suppress Cell Division Cycle Progression in a Cancer Model Cell

We propose the strategy to target the major cell division cycle transitions as a way to force cells to spend an extended amount of time undertaking each cell division cycle as they slowly re-equilibrate after a perturbation. The main cell division cycle enzyme that makes a large contribution to both the G1-S-phase transition and the metaphase–anaphase transition is the APC/C (Figure 4). We previously observed that APC/C-CDC20 Homolog 1 (CDH1) is essential for the degradation of the S-phase cyclin Clb6, which is the cyclin-E homologue, that must occur for cells to enter S-phase and initiate DNA replication [15]. It is also well established that APC/C-Cell Division Cycle 20 (CDC20) is required for the metaphase–anaphase transition by promoting the regulated degradation of the anaphase inhibitor securin, which leads to the activation of the separase enzyme that cleaves sister chromatid cohesin, which has been reviewed extensively elsewhere [16,17]. In combination, we have concluded that the APC/C should be targeted as a way to try and increase the duration of the cell division cycle because its function is critical at the points where the cell division cycle has low resilience [18]. In both cases, these cell cycle transitions executed by the same enzyme present a potential opportunity to delay mitotic progression and to increase the duration of the G1-phase, potentially inducing forced overgrowth in cells during a single cell division cycle. The APC/C is also at the center of a highly connected node within the cell, making a contribution to protein homeostasis via ubiquitin-dependent regulated protein degradation, and is also sensitive to a loss of protein folding buffering capacity [7,8,10]. Thus, the APC/C may be a potentially ”sensitized” target specifically within cancer cells suffering from an elevated load on the proteostasis machinery.

Having selected the target enzyme, the next question is as follows: what is the best-established cancer cell/chemotherapeutic drug combination to initially work with for bioactivity screening? Forced overgrowth is likely to display even more severe cytotoxic effects, especially in combination with the stress challenges the cancer cells are under when exposed to established chemotherapeutic agents. Here, we are influenced by work on bortezomib, the 26S proteosome inhibitor, using multiple myeloma cell lines, such as AMO-1 cells, where it has been established that the therapeutic index of bortezomib depends entirely upon the cyclic GMP-AMP synthase-stimulator of interferon genes (cGAS-STING) pathway, and type I interferon secretion to promote immunogenic cell death [19]. The cGAS-STING pathway responds to the presence of double-stranded DNA in the cytosol initiating the production of 2′3′-cGAMP as a secondary messenger that through activation of TANK-binding kinase 1 (TBK1) and Interferon Regulatory Factor 3 (IRF3) ultimately induces the expression and subsequent secretion of type I interferons, which has been extensively reviewed elsewhere [20,21]. Bortezomib is a first-line drug against multiple myeloma and targets the 26S proteosome as a potent inhibitor—one of the core machines in the cell essential for proteostasis and for the suppression of proteotoxic stress. This result implies that any anti-proteotoxic approach should not interfere with cGAS-STING signaling, type I interferon secretion, or immunogenic cell death that promotes tumor regression. In addition, it raises the concern that the levels of cancer cell expression of PD-L1 on the surface of the cells should be monitored.

After a review of the literature and factoring in the most successful chemotherapeutic agents with the broadest and therefore potentially the most impactful use, and where the therapeutic index depends upon cGAS-STING signaling, interferon secretion, and immunogenic cell death, we suggest initially focusing on paclitaxel as employed in the MDA-MB-231 breast cancer cell model system. Paclitaxel is FDA-approved, on- and off-label, for the treatment of more than 20 adult cancers and is one of the most widely used anti-cancer agents in the world. In a groundbreaking clinical trial published in 2014, Weaver and colleagues measured the clinically relevant dose of paclitaxel to use when working with MDA-MB-231 and CAL-51 breast cancer cell lines [22]. Briefly, they measured the amount of paclitaxel inside of the tumors of breast cancer patients that had just undergone a standard round of chemotherapy and also measured the amount of paclitaxel inside of the MDA-MB-231 breast cells that had been exposed to various titration concentrations of paclitaxel in the lab, establishing, for example, that 10 nM paclitaxel is the clinically relevant dose to work with in MDA-MB-231 cells. Since this initial success, the Weaver lab has gone on more recently to also establish the likely clinically relevant dose of eribulin to expose MDA-MB-231 cells to as well, which is in the ~1–2 nM range of eribulin [23]. In our opinion, this system established by Prof. Weaver has been one of the most informative systems to work with if the goal is to potentiate the anti-cancer activity of an agent like paclitaxel in combination with a new drug/molecule. Perhaps what is most notable about Weaver’s observations is that they have directly challenged a leading hypothesis about the mechanism of action of paclitaxel: at the level of 10 nM, it is the induction of chromosome mis-segregation events and the subsequent activation of the innate immune responses in the daughter G1-phase cells via the activation of the cGAS-STING pathway that is responsible for paclitaxel’s anti-cancer effects by promoting immunogenic cell death caused by type I interferon secretion and not, as commonly thought, complete cell cycle arrest in mitosis followed by apoptosis as induced by higher molar concentrations of paclitaxel [24]. We find these data compelling.

**Figure 5:**
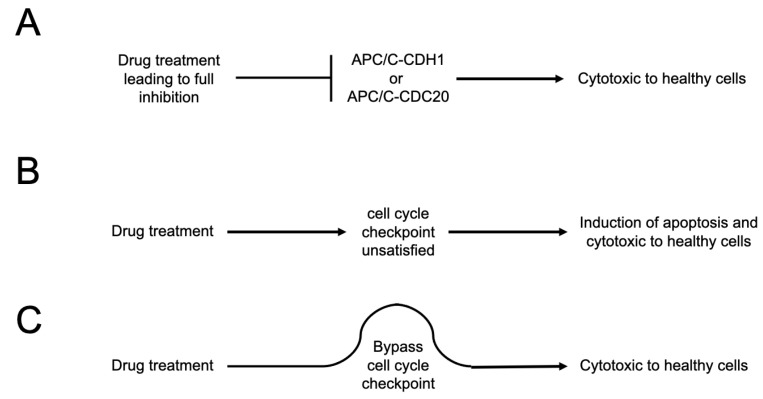
When targeting the APC/C to suppress—but not inhibit—its activity, it is important not to cause checkpoints to become unsatisfied or to prematurely satisfy them, causing a bypass of the cell cycle checkpoint. (**A**) The inhibition of APC/C activity will be a cytotoxic lethal event in healthy cells. (**B**) Causing a cell cycle checkpoint to become unsatisfied will promote apoptosis in healthy cells and will be a cytotoxic lethal event in healthy cells. (**C**) Bypassing a cell cycle checkpoint, by promoting pre-mature satisfaction of the checkpoint, will be a cytotoxic lethal event in healthy cells.

Finally, the goal we outline here is to identify and characterize molecules that increase the duration of the cell division cycle and measure the level of cell death they induce in combination with established anti-cancer agents, such as the paclitaxel system described above, or, potentially in combination with clinically approved HSP90 inhibitors (see below). However, any mechanism/molecule that suppresses APC/C activity that is intended to be developed as a therapeutic agent to work in combination with current anti-cancer drugs must be developed under several strict restraints [18]. Eight such constraints are as follows:

(i) The agent cannot block APC/C activity; it can only suppress the level of APC/C activity (Figure 5). The APC/C is an essential enzyme. Any reagent that blocks APC/C activity will be cytotoxic to healthy human cells. This is a potential hazard for CP5V, a Proteolysis-Targeting Chimera (PROTAC) molecule that promotes the degradation of the APC/C co-factor CDC20, lowering the level of CDC20 in cells [25]. Although CP5V may be useful in targeting cancer cells that depend upon the over-expression of CDC20 protein, it remains unclear whether it can decrease CDC20 levels in cancers cells relative to healthy human cells without inducing general cytotoxicity that would be deleterious [25,26].
(ii) The agent cannot cause cell cycle checkpoints to become “unsatisfied”, such as the mitotic spindle assembly checkpoint (Figure 5) [27]. In healthy human cells, long durations under which the spindle checkpoint remains unsatisfied lead to an increased level of apoptosis, a form of mitotic cytotoxicity. The most rapidly dividing population of cells in the adult human body are thought to be promyelocytes—the progenitor cells of neutrophils. Promyelocytes divide 10–100 times faster than cells in solid tumors [28]. Thus, any agent that induces mitotic cytotoxicity is very likely to severely affect promyelocytes and is thus very likely to induce neutropenia.
(iii) The agent also cannot bypass cell cycle checkpoints, again, such as the mitotic spindle checkpoint, by causing the pre-mature satisfaction of the checkpoint (Figure 5). The mitotic spindle checkpoint is essential in all dividing human cells, even cancer cells. Any bypass effect of the spindle checkpoint will induce mitotic cytotoxicity and likely will lead to neutropenia [29,30].
(iv) The agent cannot suppress the activation of the cGAS-STING innate immune response pathway caused by a chemotherapeutic agent that is thought to make a major contribution to promoting immunogenic cell death by leading to the secretion of type I interferon, such as Interferon-β (IFN-β). For example, a part of the major mechanism by which paclitaxel is thought to promote tumor regression is via the recruitment of white blood cells (leukocytes) into solid tumors where they execute immunogenic cell death [28]. How any agent that might aggravate proteotoxic stress may affect the cGAS-STING pathway, or the secretion of type I interferons, to the best of our knowledge, is not known and has not been explored.
(v) The agent cannot suppress the induction of apoptosis/necrosis caused by a chemotherapeutic agent that is thought to be a contributing factor to tumor regression. Again, how any agent that might aggravate proteotoxic stress may affect the induction of apoptosis/necrosis, to the best of our knowledge, is not known and has not been explored.
(vi) The agent should avoid the induction of the Senescence-Associated Secretory Phenotype (SASP) response in tumor cells which is known to be a contributing factor in promoting further aggressive malignancies after the completion of a round of, for example, chemotherapy [31]. Here, we have a higher concern that any agent that aggravates proteotoxic stress may elevate the SASP response because it is known that one pathway cells use to suppress proteotoxic stress is to secrete portions of the cytoplasm as exosomes [2].
(vii) The agent cannot induce the overexpression of programmed cell death-ligand 1 (PD-L1) on the surface of the target cancer cells as this will likely contribute to the suppression of immunogenic cell death that is promoted by the cGAS-STING/type I interferon secretion pathway. Here, it is already necessary to express concern about the HSP90 inhibitor pimitespib (TAS-116), which should be monitored very carefully. There is contradictory evidence with regard to HSP90 inhibition and levels of PD-L1. One study reported that HSP90 inhibition lowers PD-L1 and PD-L2 levels [32], while another study focused on pimitespib (TAS-116) reported that it causes an increase in PD-L1 on the surface of cells [33]. This may justify and/or necessitate the experimental exploration of multiple HSP90 inhibitors to investigate whether they have a similar effect (see below). Furthermore, how other agents that might aggravate proteotoxic stress may affect the induction of the expression of PD-L1 on the surface of cells, to the best of our knowledge, is not known.
(viii) The agent cannot cause elevated cell death in normal healthy and genomically stable diploid cells in order to minimize concerns about non-specific cytotoxicity and, more specifically, mitotic cytotoxicity. An agent that extends the duration of the cell division cycle alone may not cause cell death in healthy human cells, but this may not be true in the context of the co-exposure of healthy cells to an additional agent, such as a chemotherapeutic agent, which, in combination, are much more likely to induce a severe cell stress response.

If chemical agents can be identified that fit within the limitations of these criteria, then it will open new experimental avenues to explore the potential interactions between an anti-APC/C agent that suppresses its enzyme activity and an enhancer of proteotoxic stress that has regulatory approval, such as the 26S proteosome inhibitors bortezomib, carfilzomib, and ixazomib; the aggresome formation inhibitor panobinostat (that targets HDAC6); and pimitespib (TAS-116), an HSP90 inhibitor [3].

## 5. Discussion

We have outlined here for our readers the goal of trying to increase the duration of the cell division cycle. If this goal can be achieved by targeting the activity of the APC/C using molecular suppressors of its activity, we hypothesize that this injury is likely to be more cytotoxic to the aneuploid cancer cells. Currently, the small molecule that should be investigated in this regard is proTAME [34,35]. The small molecule proTAME targets the APC3 subunit where the co-factor CDH1 or CDC20 IR-tail binds, thus blocking APC/C activity. It may be possible to titrate the levels of proTAME to a low enough dose to only suppress APC/C activity instead of blocking it, to avoid inducing cell cycle arrest. In addition, the activity of proTAME can be investigated in the context of other proteotoxic drug enhancers, such as the clinically approved HSP90-inhibitor pimitespib (TAS-116) or, because of the concerns that pimitespib (TAS-116) elevates the levels of PD-L1 on the surface of cells, the pre-clinical molecule tanespimycin, to determine whether any synergistic effect between the two occurs specifically in cancer cells [3,33]. To the best of our knowledge, proTAME has not been the subject of a clinical trial; however, measurements on the pharmacodynamics/pharmacokinetics of pimitespib (TAS-116) have been reported [36,37]. Based on these studies, we estimate that a concentration of about 220–250 nM pimitespib (TAS-116), as reflected by serum blood levels, may be an appropriate amount to expose cancer cells to in the laboratory as reflection of a clinically relevant dose. Specifically, we suggest exploring exposure of MDA-MD-231 cells to proTAME in the nanomolar range in combination with pimitespib (TAS-116) in the range of about 220–250 nM over the course of 3 days and then evaluating cell death (apoptosis and necrosis) by fluorescence activated cell sorting (FACS) analyses both in the presence and absence of the clinically relevant dose of 10 nM paclitaxel. Such experiments could be complemented by time-lapse video microscopy to observe any effects on the timing of the cell division cycle and may uncover a novel approach to attack cancer cells that aims to exploit proteotoxic stress that is unique to aneuploid dividing cancer cells.

## Abbreviations

The following abbreviations are used in this manuscript:

CDC	Cell division cycle
APC/C	Anaphase-promoting complex/cyclosome
FDA	Food and Drug Administration
HDAC6	Histone Deacetylase 6
IFN-β	Interferon-β
cGAS-STING	Cyclic GMP-AMP synthase-stimulator of interferon genes
CDC20	Cell Division Cycle 20
CDH1	CDC20 Homolog 1
TBK1	TANK-binding kinase 1
IRF3	Interferon Regulatory Factor 3
PROTAC	Proteolysis-Targeting Chimera
PD-L1	Programmed cell death-ligand 1
PD-L2	Programmed cell death-ligand 2
SASP	Senescence-Associated Secretory Phenotype
